# Genetic regulation of parasite infection: empirical evidence of the functional significance of an IL4 gene SNP on nematode infections in wild primates

**DOI:** 10.1186/1742-9994-8-9

**Published:** 2011-04-18

**Authors:** Dagmar Clough, Peter M Kappeler, Lutz Walter

**Affiliations:** 1 Behavioral Ecology and Sociobiology Unit, German Primate Center, Kellnerweg 4, 37077 Göttingen, Germany; 2 Dept. of Anthropology / Sociobiology, University of Göttingen, Kellnerweg 6, 37077 Göttingen, Germany; 3 Primate Genetics Laboratory, German Primate Center, Kellnerweg 4, 37077 Göttingen, Germany

## Abstract

**Background:**

Susceptibility to parasite infection affects fitness-related processes, such as mate choice and survival, yet its genetic regulation remains poorly understood. Interleukin-4 (*IL4*) plays a central role in the humoral immune defence against nematode parasite infections, inducing IgE switch and regulation of worm expulsion from the intestines. The evolutionary and functional significance of single nucleotide polymorphisms (SNPs) in *IL4*-genes is known, yet empirical information on the effect of *IL4 *SNPs on gastro-intestinal infections is lacking. Using samples from a population of wild red-fronted lemurs (*Eulemur fulvus rufus*, Primates: Lemuridae), from western Madagascar, we explored the association of *IL4*-gene promoter polymorphisms with nematode infections and investigated a possible functional role of the *IL4 *polymorphism on male reproductive success.

**Results:**

Using sequence analyses of lemur DNA we detected a new SNP in the *IL4 *gene promoter area. Carriers of the genotype T/T showed higher nematode infection intensities than individuals of genotypes C/T and C/C. Genetic population analyses using data from more than 10 years, suggested higher reproductive success of T/T males than expected.

**Conclusions:**

Our results suggest a regulatory effect of an *IL4 *gene promoter polymorphism on the intensity of parasite infections in a natural population of red-fronted lemurs, with a seemingly disadvantageous genotype represented in low frequencies. Long-term population analyses, however, point in the direction of a negative frequency-dependent association, giving a fitness advantage to the rare genotype. Due to low frequencies of the genotype in question conclusive evidence of a functional role of *IL4 *polymorphism cannot be drawn here; still, we suggest the use of *IL4 *polymorphism as a new molecular tool for quick assessment of individual genetic constitution with regard to nematode infection intensities, contributing to a better understanding of the actual components of the immune response that mediate protection against gastro-intestinal parasites.

## Background

Parasite infections impose high costs on both human and animal populations, increasing morbidity and mortality, particularly in hosts under ecological stress [1-3]. Understanding the genetic regulation of parasite resistance in natural population is of major importance for understanding host-parasite evolution and host sexual selection processes. In the past, great effort has been devoted to study major histocompatibility complex (MHC) diversity and compatibility as a key element of genetic regulation of parasite resistance and a potential driving force in sexual selection processes, respectively [4-12]: host genetic variation can be promoted by parasites through frequency-dependent selection on advantageous resistance alleles [10,13], and individuals that are heterozygous at the MHC are expected to have a selection advantage and to be better capable of combating a variety of infectious agents than MHC homozygotes [5,7,14]. Additionally, some studies report correlations between parasite resistance and individual heterozygosity that are explained by reduced fitness values of homozygous individuals for traits that are controlled by directionally dominant loci [13,15,16]. Still, there is accumulating evidence that individual heterozygosity often appears to be a weak predictor of parasite infection and the importance of specific alleles of candidate genes in regulation of parasite infection has been suggested [15,17]. In this respect, cytokine genes such as interleukins are natural candidates due to their major regulatory role in helminth parasite susceptibility [18], and recently Fumagalli and colleagues [19] highlighted their evolutionary significance as a target of balancing selective processes.

Immunity to helminth parasite infections is mainly mediated by CD4+ T-helper 2- (T_H_2) lymphocytes with promotion of T_H_2 immune responses (humoral immune responses) being dependent on the cytokine interleukin-4 (IL-4) [18]. IL-4 not only induces and sustains T_H_2 responses and suppresses T_H_1 responses, but also initiates immunoglobulin (Ig) isotype switching to IgE, which plays an essential role in anti-parasite immunity [20]. Evidence mainly from human diseases is accumulating that single nucleotide polymorphisms (SNPs) in the promoter region of the interleukin 4 gene (*IL4*) affect its transcription, resulting in altered IL-4 protein levels and, hence, in either higher or lower IgE titres [21-24]. In this way, *IL4 *SNPs can effectively influence the intensity of various infections [22,25-30], including enteric pathogens [31-34]. Still, despite its key role in the regulation of parasite infections and resulting evolutionary significance of *IL4 *polymorphisms [19], empirical evidence of the importance of *IL4 *on the intensity of parasite infections in natural population is lacking.

This study integrates field parasitology and population genetics to investigate the functional significance of polymorphisms in the *IL4 *gene on gastro-intestinal infections in a wild non-human primate: the red-fronted lemur (*Eulemur fulvus rufus*).

Red-fronted lemurs live in small multi-male, multi-female groups of 5-12 individuals with an even or slightly male-biased adult sex ratio [35,36]. Reproduction is highly seasonal with only one mating period per year. During this three-to four-week period, females are in oestrus for approximately one day and mate promiscuously with several males [37,38], resulting in a very low male mating skew within a group [39]. In contrast, reproductive skew is very high as male reproductive success is positively correlated with male dominance rank [40]. Variation in parasite infection of the red-fronted lemur population on which this study is based on has been investigated in detail and is known to differ significantly among individuals but not between males of different rank [41].

The specific objectives of our study were: (1) to identify promoter SNPs in the *IL4 *gene of the red-fronted lemur, (2) to associate both the respective *IL4 *SNP genotypes and a measure of individual heterozygosity with intensities of nematode infections, (3) to identify a possible functional role of the *IL4 *alleles in selective processes by exploring long-term fitness consequences between males of different genotype constitutions. We expected frequency-dependent selection to result in a higher frequency of genotypes, which provide the best resistance to parasites [10]. Further, if *IL4 *does obtain a functional role in selective processes, we predicted a fitness advantage of individuals with a beneficial *IL4 *genotype.

## Results

### *IL4 *promoter polymorphism in red-fronted lemurs

**Figure 1:**
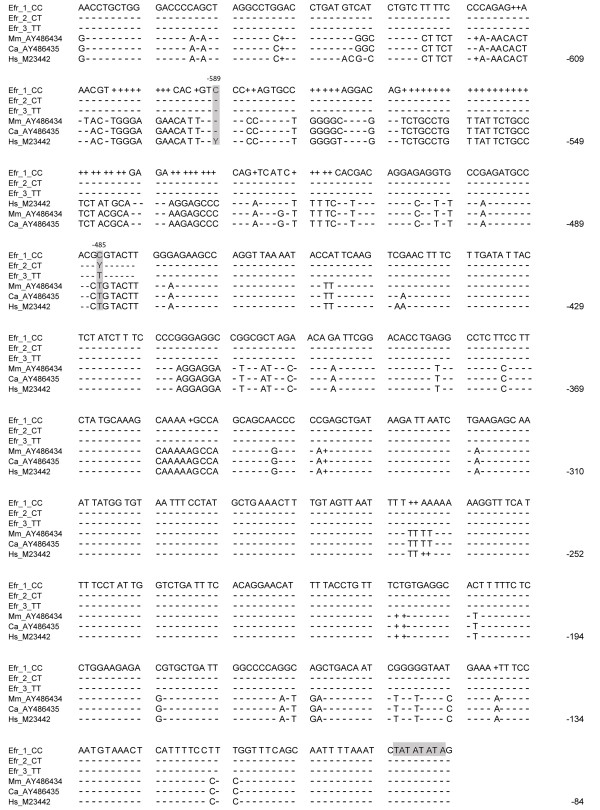
***Eulemur fulvus rufus IL4 *promoter sequence**. Alignment of *Eulemur fulvus rufus IL4 *promoter sequence (GenBank accession GQ221019) with published sequences of human [Hs, 70], *Macaca mulatta *[Mm, 71] and *Cercocebus atys *[Ca, 71]. Highlighted are the lemur -485C/T SNP, the human -589C/T SNP, and the TATA box. Gaps introduced to maximise similarity are marked by "+". Nucleotides identical to the lemur sequence (Efr_1_CC) are shown by dashes. Nucleotide numbering is based on the human sequence.

After sequencing a 528 bp fragment of the *IL4 *promoter region of the red-fronted lemur, we identified a C/T polymorphism at position -485 bp upstream of the transcription start site (Figure 1). The -485 C/T was the only SNP found within this part of the promoter sequence. All possible genotypes (C/C, C/T, T/T) were present in the total lemur population studied between 1996 and 2007 (N = 64 individuals) with the following frequencies: C/C: 51.6% (n = 33 individuals), C/T: 37.5% (n = 24), T/T: 10.9% (n = 7). In a subset of this dataset, which was used for combined parasitological and genetic analyses (see below; yr 2007, n = 24 individuals) frequency distribution was C/C: 50.0% (n = 12 individuals), C/T: 37.5% (n = 9), T/T: 12.5% (n = 3). Frequency distribution of genotypes did not deviate from a distribution expected under Hardy-Weinberg equilibrium (Fisher's exact test, p = 0.86, df = 2).

### Parasite infection intensities and association with *IL4 *gene promoter polymorphism

During the study period in 2007, all lemurs of both sexes (13 males, 11 females) were parasitized by at least three helminth morpho-species [see Ref 42 for more details] with most prominent infections with the nematode species *Lemuricola vauceli *and *Callistoura *sp. Animals showed no signs of clinical significance during the period of the study that could be linked to acute parasite infections (intense behavioural observation were conducted on the same animals for other purposes, see Refs [41,42]). Intensities of individual nematode infections ranged from 0 to 3300 eggs per faecal sample with median infection intensity of 100 eggs/g faeces.

**Figure 2:**
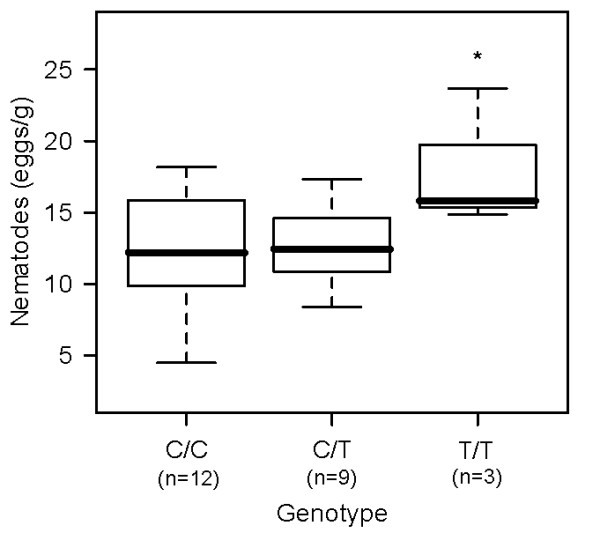
**Parasite infection intensity and *IL4 *genotype**. Box plots of nematode infection intensities between individuals of different genotypes (n = 24). Response variables are depicted as sqrt-transformed data. * denotes significance at p < 0.05

The intensity of nematode infections differed significantly between *IL4 *genotypes (Figure 2, Table 1): individuals of genotype T/T had higher parasite egg outputs than individuals of the more frequent genotypes C/T and C/C (t_24,2 _= 2.20, p _CC-TT _= 0.04, p _CT-TT _= 0.04).

**Table 1:** General linear mixed effect model of nematode infection intensity

Term	df	χ²-value	P-value	Effect direction
**IL4**	**2**	**14.14**	**< 0.001**	**T/T > C/C, C/T**
sex	1	0.0091	0.924	No effect
season	2	< 0.001	0.99	No effect

Significant terms are highlighted in bold. P-values were estimated by comparison with reduced models not containing the term in question (likelihood-ratio test), n = 24.

### Individual heterozygosity

**Figure 3:**
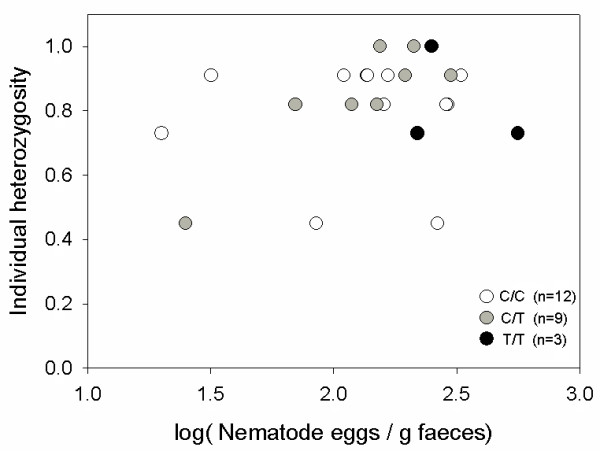
**Parasite infection intensity and individual heterozygosity**. Nematode infection intensities are not associated with individual heterozygosity (r = 0.05, p = 0.8, n = 24). Genotypes are displayed in different shades.

Mean individual heterozygosity was 0.79 (± 0.17) and did not correlate with individual nematode infection intensities (r = 0.05, p = 0.8, n = 24, Figure 3).

### Fitness consequences of different *IL4 *genotypes

**Figure 4:**
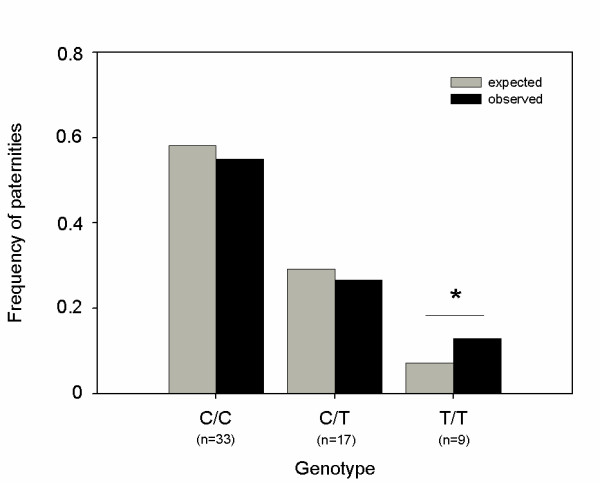
**Distribution of paternity share between different *IL4 *genotypes**. Observed frequencies of paternities differed significantly from expected patterns in animals of genotype T/T (n = 59).

Observed reproductive success (ORS) of males of different genotypes ranged from 0 to 100% and differed significantly from expected values (Expected reproductive success ERS). Individuals with the genotype T/T sired significantly more offspring than expected (ORS_TT _= 13%; ERS_TT _= 7%; χ² = 4.47, p < 0.05, df = 1; n _T/T _= 9; Figure 4), whereas observed paternity success in individuals of genotype C/C (n _C/C _= 33) and C/T (n _C/T _= 17) did not deviate from expectations (Figure 4).

## Discussion

In this study, we combined information on individual parasite infection intensities, immune-genetic constitution, and long-term parentage patterns to investigate a potential regulatory and functional role of *IL4 *SNPs on enteric parasite infections in a wild primate population. We detected a polymorphic site at the *IL4 *gene promoter region at position -485 bp that was not identical to known and functionally relevant *IL4 *promoter polymorphisms in humans or other non-human primates [29,43-45]. We investigated individual parasite infection intensities with regard to different *IL4 *genotypes and found evidence for differential association of the three groups of genotypes with nematode infection intensities: animals carrying the rare genotype (T/T) had higher nematode egg outputs than carriers of the genotype C/C and C/T. However, contrary to our expectations, long-term population analyses indicated a disproportionately higher reproductive success of genotype T/T individuals.

### IL4 promoter polymorphisms and parasite infections

In general, promoter SNP-modulated gene transcription can lead to differential activity of a gene and is frequently based upon altered transcription factor binding properties at the site of the mutation [46]. Studies *in vivo *and *in vitro *on a well-known human C/T polymorphism (-589CT) showed that individual genotypes bearing the binding site of transcription factor nuclear factor of activated T-cells (NFAT) [47]) had altered transcription rates of *IL4 *mRNA resulting in differential IL-4 production [23,24,27,46,48]. Experimental evidence for altered transcription rates cannot be provided here. Yet, we suggest that the -485C/T SNP detected in red-fronted lemurs affects *IL4 *gene transcription in a similar way: decreased *IL4 *mRNA and thus decreased IL-4 protein levels in T/T individuals could thus contribute to higher nematode infection intensities as IL-4 is known to play an important role in enteropathic expulsion of nematode worms and increases mucosal permeability after infections [31-34]. Alternatively, the observed association of *IL4 *polymorphism and infection intensities could be due to linkage disequilibrium of *IL4 *with other genes such as *IL13 *and *IL5*, which in humans are located just 12.5 kb and 132 kb upstream of *IL4 *and are also key T_H_2 cytokines [31].

### Individual heterozygosity and parasite infections

Mean individual heterozygosity in the study population was 0.79, which suggests that the population was not subject to inbreeding. In line with results from other studies [15,49] and our expectations, the present findings did not confirm a relationship between individual heterozygosity and parasite infection intensity, suggesting that enteric parasite infection in lemurs is associated with one specific genotype of a candidate-gene rather than with heterozygosity per se. The power of this result is certainly constrained by the ability to estimate overall heterozygosity by use of a limited number of microsatellite markers [50,51]. However, the usage of 11 microsatellite markers is comparable to most previous studies in vertebrates [13,15,49,52].

### Functional significance of the *IL4 *polymorphism

Significant parasite resistance is generally thought to be beneficial in terms of individual fitness [10,53]. We expected individuals with the more common genotypes (C/C and C/T), characterized by low parasite infection levels, to be superior to T/T individuals, which had highest infection levels. However, long-term paternity analyses indicated a disproportionally higher reproductive success of these T/T males, although the genotype was only found in 11% individuals of the total study population. This indication of a negative frequency-dependent association (low frequency - high reproductive success) is contradictory to other studies conducted on birds [54,55] and fish [56].

A potential explanation may be found in the counterbalancing function of IL-4. A regulatory polymorphism in the *IL4 *promoter can influence the activity of the cytokine and thus the balance of the T_H_1/T_H_2 ratio. Such a balancing function results either in an increased T_H_1 response (low IL-4 level), potentially advantageous when individuals are confronted with intracellular pathogens such as viruses or phagocytised bacteria, or an intensified T_H_2 response (high IL-4 level), required when individuals are affected by extra-cellular parasites such as nematode worms [48]. This suggests that the *IL4 *promoter polymorphism may be subject to balancing selection. Imbalanced T_H_1/T_H_2 ratios are known to be responsible for the lepromatous form of leprosy and influence susceptibility to allergy in "parasite-free" industrialized areas in human [57,58]. With regard to our study population, it is likely that an IL4-regulated and intensified T_H_1 response targeting microparasites (not measured in this study) could provide an explanation for the disproportionately higher reproductive success of these animals. Yet, although this scenario is a plausible explanation for the significantly higher long-term reproductive success of T/T individuals, the *IL4 *gene is certainly only one of many factors affecting variance in male reproductive success in a wild lemur population [40]. Additionally, increased levels of nematode infection might not be a crucial aspect in selection processes of this particular population. Analyses on determinants of parasite infection in red-fronted lemurs published elsewhere [41] show, for example, that the intensity and species richness of nematode infections has no effect on social rank or mating success. In addition, most prominent nematode species found in this study are not considered as highly pathogenic [42] when compared to tissue migrating nematodes that may also contribute to anaemia and secondary bacterial disease [59].

## Limitations of the study

This is the first study exploring the significance of IL4 polymorphisms simultaneously in several groups of a wild primate species, which involves an unusually large logistic effort. There are two major caveats in this study, which ask for a cautious interpretation of our results. First, studying natural populations of primates is often associated with the drawback of working with small sample sizes - in particular when compared to laboratory-based studies with murine models - and in our study, the number of individuals per individual genotype, in particular for the most interesting genotype, was very low (year 2007: n_T/T _= 3). To minimise bias, we used conservative statistical methods that accounted for non-independent data and unbalanced designs. Second, this is a genetic association study and lacks in-vitro and in-vivo evidence of T_H_1/ T_H_2-driven responses influenced by promoter polymorphisms. In particular, this leads to a speculation that the T/T genotype has diminished expression of a T_H_2 immune response, contributing to the higher nematode egg excretion levels observed. Recent functional studies (particularly in mice) have established the link between T_H_2 levels and parasite infection intensities in detail [18,20].

These two caveats hamper firm conclusions for the time being. While our results suggest interesting patterning of an *IL4 *polymorphism and its effect on intestinal parasite infection in wild primates that can be explained biologically, these caveats will need to be addressed in future studies to improve the data basis and our mechanistic understanding of the observed pattern.

## Conclusions

We detected a novel C/T polymorphism at position -485 bp in the promoter region of the *IL4 *gene in a wild primate population. The association of this polymorphism and inter-individual variability in nematode infection encountered in this lemur population indicated that carriers of a rare genotype had higher nematode infection intensities than carriers of the more common genotypes. However, against our expectations, long-term paternity analyses indicated above-average reproductive success of the former.

Due to low frequencies of the particular *IL4 *genotype in our study population final conclusions on the functional role of *IL4 *polymorphism cannot be drawn yet. Still, the methodological approach used in this study may contribute to a better understanding of the actual components of the immune response that mediate protection against helminth parasites and is recommended for further studies. If similar patterns are found in other natural study systems, the analyses of *IL4 *promoter SNPs could provide an efficient scoring system for susceptibility to helminth infections.

## Methods

### Study site and sample collection

Data were collected at the study site of the German Primate Center (DPZ) in Kirindy Forest, western Madagascar. Detailed description of the study site can be found in Sorg et al [60]. Between 1996 and 2007, adult red-fronted lemurs of the study population belonging to four social groups (groups A, B, F, J) living within a 60 ha study area have been regularly captured and marked individually with unique nylon or radio collars (in total: 48 males, 16 females). From these animals, small tissue samples were routinely taken and stored in 70-90% ethanol for DNA extraction [40]. During a 3-month study period between April and July 2007, a total of 299 faecal samples were collected weekly from each individual of the current population (13 males, 11 females) for parasitological analyses. Individually assigned samples were taken immediately after defecation, stored in labelled vials containing 10% buffered formalin. All samples were transported to DPZ laboratories, Germany, for analyses. Ethical approval for this study was not necessary because our research was not experimental. Study animals were not subjected to experimental manipulations to obtain any of the data presented in this paper. All field work was carried out following the guidelines of the American Society of Mammalogists. The capture of study animals, which was used to obtain tissue samples, was authorized by the Malagasy Ministère de l'Environnement et des Eaux et Forêts.

### Genetic analyses

#### *IL4 *promoter sequencing

DNA was isolated from tissue samples of all 64 individuals using QIAamp^® ^tissue kits (Qiagen). A fragment of the *IL4 *gene promoter region was amplified using primers: forward 5'-CATACGAACCTGCTGGGAC-3' and reverse 5'-CAATCAGCACGTCTCTTCCA-3'. Hot start PCR was carried out in a total volume of 30 μl with 10 pmol of each primer, 166 μM dNTPs, and 2U Taq DNA polymerase. Amplification was performed according to the following protocol: 5 min at 92°C, 45 cycles of 92°C for 1 min, 58°C for 1 min and 1 min at 72°C, and final elongation for 5 min at 72°C. PCR products were purified with the Millipore DNA purification kit (Millipore, Schwalbach, Germany) and sequencing was performed in both directions with a BigDye terminator sequencing kit (Applied Biosystems, Darmstadt, Germany) in an ABI 3130 × l automated capillary sequencer (Applied Biosystems) with same primers as mentioned above. Individual *IL4 *sequences were aligned and examined for occurrence of SNPs using the biological sequence alignment editor BioEdit 7.0.9 [61]. The newly discovered SNP at position -485 bp upstream the transcription start has been submitted for publication in dbSSNP data base [ss142460308]. The *IL4 *promoter sequence is stored in the DDBJ/EMBL/Genbank database, accession number GQ221019.

#### Individual heterozygosity

Controlling for a potential heterozygosity effect, we analysed the effect of multi-locus marker heterozygosity (MLH) on parasite infection [52,62]. Animals from the 2007 population (n = 24, see above) were typed at eleven highly variable microsatellite markers [see [40,63]] and MLH was determined as the proportion of typed loci for which an individual was heterozygous [64]. Allele frequency analyses conducted in CERVUS 2.0 confirmed that none of the markers deviated significantly from Hardy-Weinberg-equilibrium.

#### Reproductive success

Exploring a functional role of different *IL4 *genotypes on male reproductive success, long-term reproductive success of all males abundant in all four social groups of the study population from 1996 to 2007 (n = 48) was assessed via parentage analyses. During this time period, 59 offspring of which fathers could be identified genetically were born in the study population. Detailed methods on microsatellite-based paternity analyses as well as paternities are published elsewhere [40]. As both dominant and subordinate males mate multiply with females and there is only very little mating skew within a social group [39], we predicted expected male reproductive success (ERS) based on the distribution of males with respective genotypes (C/C, C/T, T/T) per social group, year, and the number of offspring born to the group in the respective year. Based on the result of paternity analyses, we then calculated observed male reproductive success (ORS) per genotype and year.

### Parasitological analyses

Faecal parasites samples were processed using a modified form of the formalin-ethyl-acetate sedimentation technique as described by Ash and Orihel [65], and detailed methods can be found elsewhere [42]. Wet mounts were prepared using 20 mg faecal sediment, analyzing individual samples for intestinal helminth parasites (nematodes, cestodes, trematodes). Larvae and adult stages found in faecal samples were used for identification of morpho-species. In cases were adult worms were available determination to species level was possible. Due to very low numbers of trematode and cestode parasitic stages (see Ref. [42] for details), we focussed our analyses of helminth parasites on nematodes. Results on nematode egg morpho-types were extrapolated to 1 g faecal sediment (x50). We used parasite infection intensity (number of eggs) as a measure of parasite infection levels. Due to parasite-specific variation in egg shedding, there has been some discussion about the reasonable use of faecal egg counts as a measure of infection intensity [66,67]. We accounted for natural occurring variation in parasitic excretions by using monthly medians of faecal egg/cyst counts per individual and pooling the data for all nematode infections to generate the response variables for statistical analyses.

### Statistical analyses

We used monthly means of faecal egg counts per individual as a measure for natural occurring variations in parasitic excretions [66,67]. We modelled differences between individuals of different genotypes using a linear mixed model approach (lmer in R [68]). As both response variables showed high degrees of overdispersion (see Ref. [41] for details), which could not be improved by applying a GLMM (link = log) with quasi-error structure as advised for use of parasitological data [69], we used square-root transformed data on individual means (GLMM, link = identity). After transformation distributions of response variables were no longer different from the normal distribution. Homogeneity of variances was checked using residual plots. Residuals of all models were constant and normally distributed as checked by Q-Q plots. Non-independence of repeated measurements per individual as well as potential group-specific variation (e.g. due to behavioural differences or habitat use)was accounted for by incorporating individual nested in group (four levels: social group A, B, F, J) [69]. As we know from earlier analyses (published elsewhere, see Ref [41]) that season and sex can have a significant effect on the intensity of parasite infection, we included both factors as fixed effect covariates in the model. Full model structure was: Response = nematode infection intensity; Fixed effect factor = genotype; Fixed effects covariate = sex, season (pre-, mating, post-season as defined in Ref. [41]); Random effects = individual, group. Model simplification was conducted by step-wise removal of non-significant parameters. Nested models with different fixed effects were compared using likelihood-ratio tests with ML estimation [70], which was also used to confirm lack of contribution of eliminated variables. P-values for mixed models were estimated using Markov Chain Monte Carlo (MCMC) simulations [71].

Relationships of individual heterozygosity (MLH) and nematode infection intensities (log-transformed) were assessed using Spearman rank correlation analyses. Observed and expected frequencies of paternities per offspring per male were compared between genotypes with χ² test statistics. All statistical analyses were performed with software R (Version 2.8.1 [72]) and the significance level was set at 0.05.

## Competing interests

The authors declare that they have no competing interests.

## Authors' contributions

DC, PMK and LW designed the study. DC took the leading role in writing the manuscript, PMK and LW contributed to the manuscript. All authors read and approved the final version of the manuscript.
